# Supplementary material for: Improving the Efficiency of Geographic Target Regions for Healthcare Interventions
Source: Int J Environ Res Public Health. 2022 Nov 9;19(22):14721. doi: 10.3390/ijerph192214721 (PMC9691133; doi:10.3390/ijerph192214721)
Supplement: Supplementary file 1 [file ijerph-19-14721-s001.zip › ijerph-1964712-SM.pdf]

Supplementary Material

Contents

Full range of values of polygons and polygon fragments underlying Figures 2 and 3 in the main text

Sensitivity to the number of polygons created per minimal unit within the STA

Application of the STA to modelled, or smoothed, risk surfaces

Supplementary material references

Full range of values of polygons and polygon fragments underlying Figures 1 and 2 in the main text

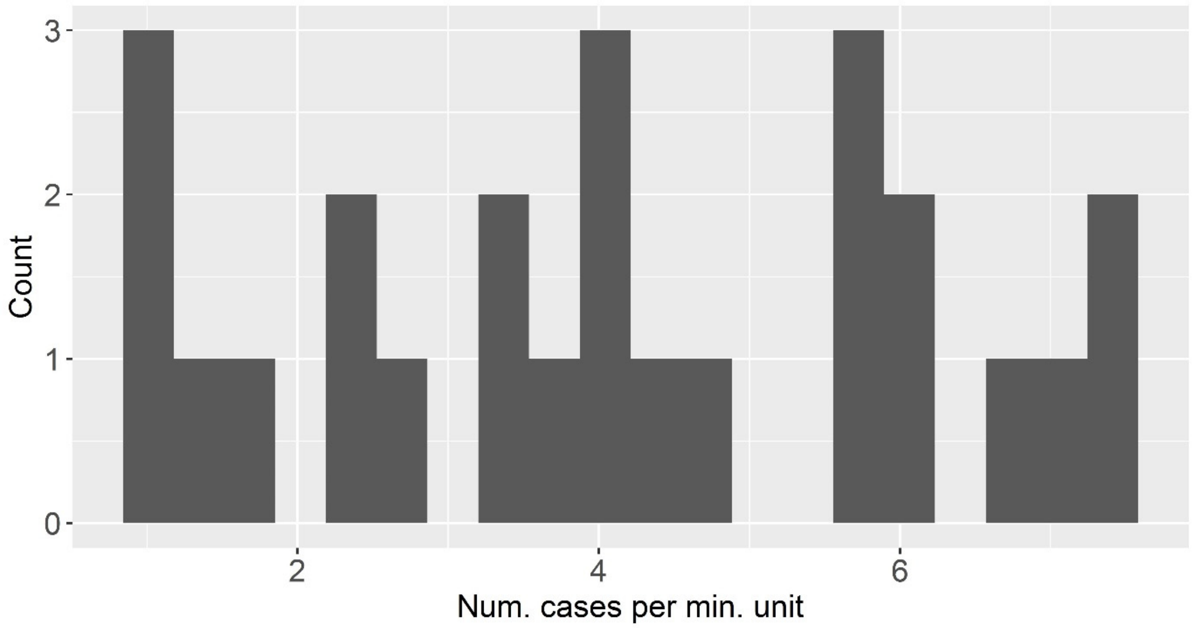

Figure S1. Full range of values among polygons in Figure 2, main text.

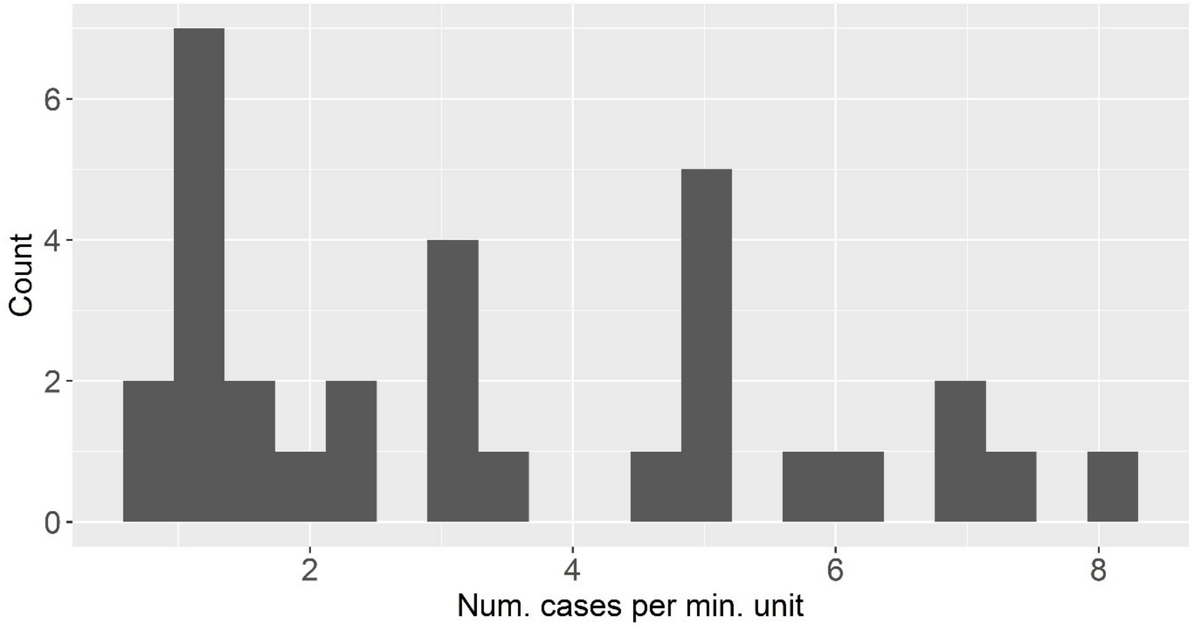

Figure S2. Full range of values among polygons and polygon fragments in Figure 3, main text.

Sensitivity to the number of polygons created per minimal unit within the STA

Fig. S3a. shows mean percentage increase in efficiency curves for the distance-weighted STA applied *with* splitting, produced while incrementing the number of polygons created per minimal unit within the STA between 1 and 25. The three curves shown correspond to target case percentages of 25%, 50% and 75%; they are each based on averaging results across 100 simulated datasets. Corresponding curves for the value-weighted STA are shown in Fig. S3c. The curves in Figs. S3a and S3c demonstrate that, in general, targeting efficiency increases as more polygons are created per minimal unit within the STA, but that this increase gradually decrease in magnitude before plateauing at zero when more than approximately 15 or 10 polygons are created per minimal unit, respectively. The plateaus are more plainly observed in Figs. S3b and S3d, which show curves derived through averaging those such as are shown in Figs. S3a and S3c, but for target case percentages ranging between 1 and 100.

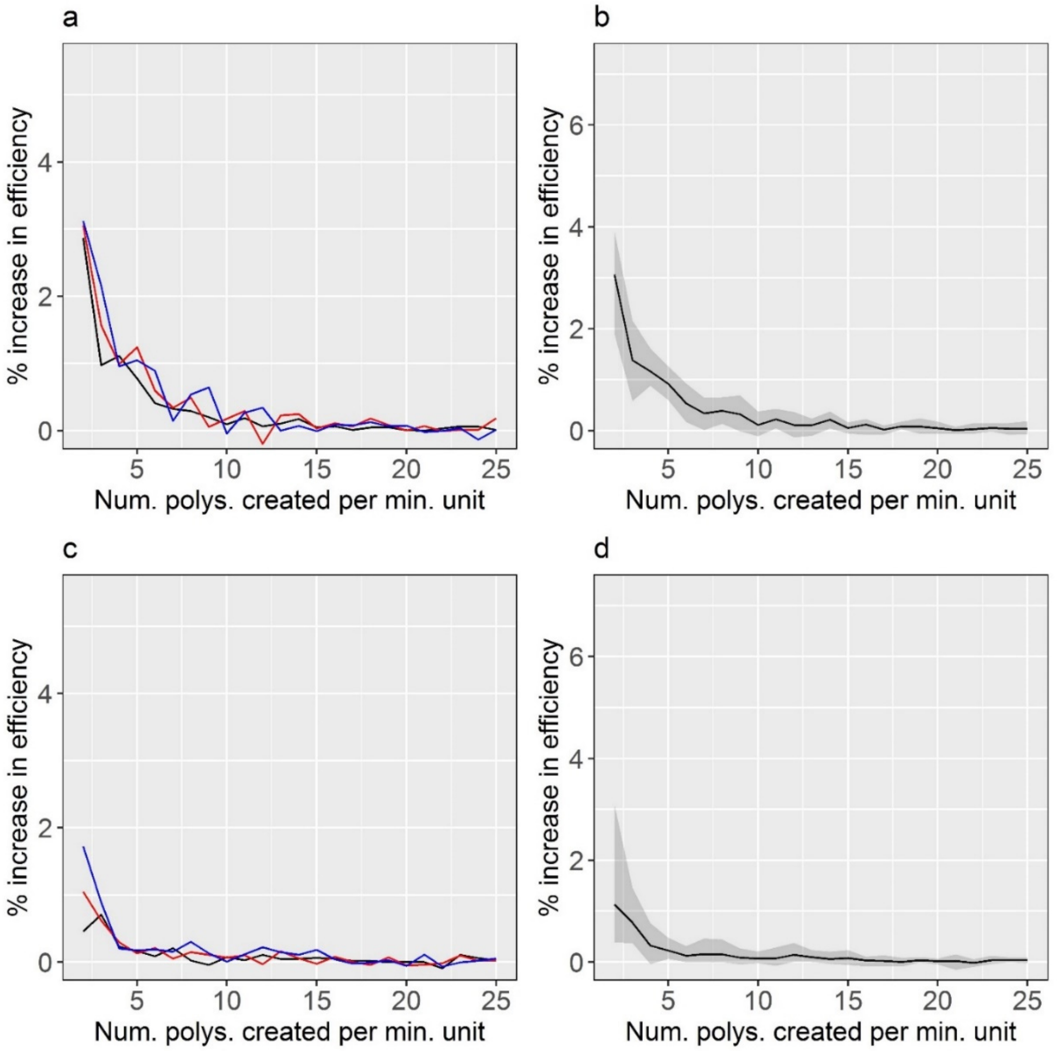

Figure S3. (a,c). Curves plotting the mean percentage increase in efficiency for the distance- and value-weighted STAs, respectively, against the number of polygons created per minimal unit (varying between 1 and 25). Three curves are shown in each panel: these represent target case percentages of 25%, 50% and 75%; they are based on averaging results across 100 simulated datasets. **(b,d).** Mean percentage increase in efficiency curves derived through averaging curves such as those shown in (a,c), across target case percentages ranging between 1 and 100. In (b,d), shaded regions represent 95% quantile bands based on results for the 100 simulated datasets and the different target case percentage curves.

Application of the STA to modelled, or smoothed, risk surfaces

This section describes the application of the STA to a smoothed risk surface. To illustrate, using a recently proposed smoothing technique – the Overlay Aggregation Method (OAM; [S1]) – we produced several maps of varying degrees of smoothness of the simulated dataset described in the main text (Figs. S4a, S4d and S4g). Briefly, OAM involves combining multiple, aggregate-level disease maps of a given minimal-resolution dataset to obtain a single, minimal-resolution map. Following [S1], we used the publicly available software AZTool [S2,S3] to create aggregate-level zonations for use within OAM. Then, to produce maps with different degrees of smoothing, we specified target denominator sizes of 4, 8 and 16 minimal units, and associated minimum threshold denominator sizes of 3, 6 and 12 minimal units, within AZTool. For each pair of target and minimum threshold denominator sizes, we created 100 different zonations. Finally, to each smoothed map we applied both the distance- and value-weighted STAs, with splitting, based on a target case percentage of 25%. Resulting portfolios of TRs are shown in Figs. S4b, S4e and S4h (for the distance-weighted STA) and Figs. S4c, S4f and S4i (for the value-weighted STA). As might have been expected, these results demonstrate that the degree of difference between the distance- and value-weighted results is inversely proportional to the degree of smoothing initially undertaken.

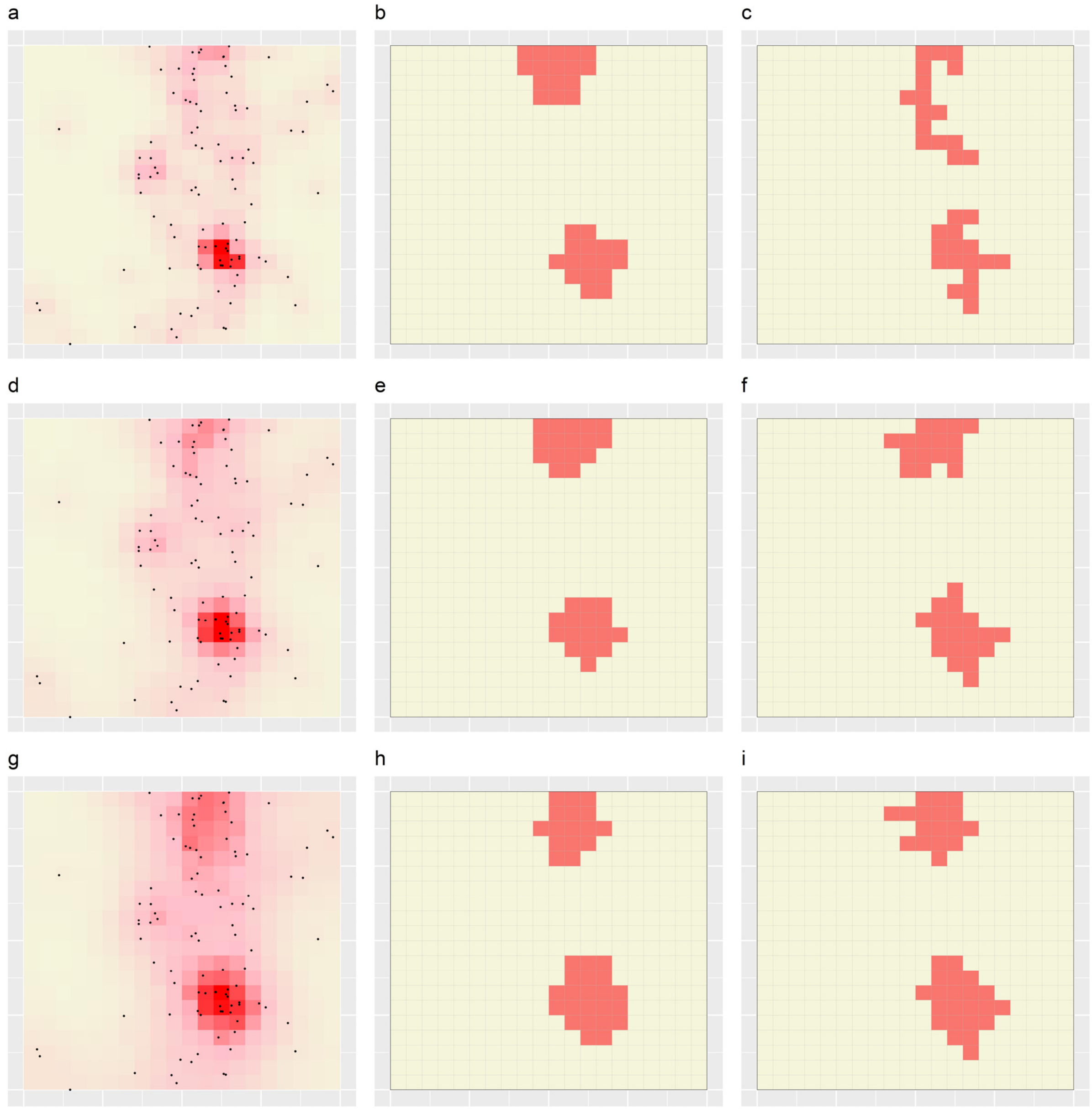

Figure S4. Illustration of the STA's application to smoothed risk surfaces. (a,d,g). Smoothed surfaces produced through applying the smoothing technique OAM to a simulated dataset with different degrees of smoothing. In (a,d,g), the unit mapped is a weighted mean number of cases per minimal unit. **(b,e,h).** TRs delineated through applying the distance-weighted STA without splitting to the surfaces in (a,d,g). **(c,f,i).** TRs delineated through applying the value-weighted STA without splitting to the surfaces in (a,d,g). In all panels, grid lines represent the set of minimal units specified for both OAM and the STA. In (a,d,g), black dots depict the locations of the simulated cases. In each of (b-c), (e-f) and (h-i), shaded regions comprise ≤ 25% of the relevant smoothed case numbers.

Supplementary material references

[S1] Tuson M, Yap M, Kok MR, Boruff B, Murray K, Vickery A, Turlach BA, Whyatt D. Overcoming inefficiencies arising due to the impact of the modifiable areal unit problem on single-aggregation disease maps. *Int J Health Geogr*. 2020. 19:40. DOI: https://doi.org/10.1186/s12942-020-00236-y

[S2] Cockings S, Harfoot A, Martin D et al. Maintaining existing zoning systems using automated zone-design techniques: methods for creating the 2011 Census output geographies for England and Wales. *Environ. Plan A*. 2011; 43(10):2399-418. DOI: https://doi.org/10.1068%2Fa43601

[S3] Martin D. Extending the automated zoning procedure to reconcile incompatible zoning systems. Int J *Geogr Inf Sci*. 2003; 17(2):181-96. DOI: http://dx.doi.org/10.1080/713811750
